# Impact of a WeChat-based intervention on nurses knowledge and practice in peripheral venous catheter insertion

**DOI:** 10.1038/s41598-025-03430-9

**Published:** 2025-05-25

**Authors:** Ai-zhen Hu, Xiao-ying Yin, Chen Luo, Liang-ai Lu, Hong-li Lai, Lin Liu

**Affiliations:** 1 Jiangxi Provincial People’s Hospital, The First Affiliated Hospital of Nanchang Medical College, Nanchang, 330006 People’s Republic of China; 2 The Second Affiliated Hospital of Nanchang University, Nanchang, 330008 People’s Republic of China

**Keywords:** PIVC, Nurses, Knowledge, Practice, WeChat, People’s Republic of China, Diseases, Health care, Health occupations

## Abstract

Peripheral intravenous catheter (PIVC) insertion is a constituted a routine invasive clinical procedure predominantly performed by nursing staff in hospital settings. While essential for short-term intravenous therapy administration, this intervention carried inherent risk of procedure-related complications due to its invasive characteristics. Consequently, enhancing nurses’ clinical knowledge and operational competencies in PIVC placement and maintenance emerged as a critical determinant of patient safety and therapeutic outcomes. The aim of this study was to investigate the impact of knowledge and behaviour on nurses’ implementation of peripheral intravenous catheters via a WeChat push-based service. The findings provided empitical evidence to guide the formulation of targeted nurse education programs and optimize training strategies, thereby contributing to healthcare service quality improvement and enhanced patient safety protocols. This study enrolled 144 nurses from a tertiary general hospitalin China, conducted between March and September 2024. To align with clinical practices in China and ensure data validity, the research team revised the survey instruments based on three national standards: the Chinese version of the Standard Guide to Practice Standards for Intravenous Infusion Therapy (2021 edition), the Standard of Practice for Intravenous Therapy Nursing Techniques (WS/T 433-2023), and the Clinical Nursing Practice Guidelines for Common Complications of Intravenous Catheters, supplemented by a systematic review of relevant domestic literature. For the intervention group, targeted educational content on PIVC placement was developed and delivered through the WeChat platform. Participants received 2–3 concise messages teice daily over a 10-day period. Both the intervention and control groups underwent standardized assessments of PIVC knowledge and clinical skills at three time points: preintervention, postintervention, and 4-week follow-up. This study enrolled 123 nurses, with 64 assigned to the intervention group and 59 to the control group. At baseline, no significant difference existed in mean knowledge (4.38 vs. 4.53, *P* > 0.05) or practice scores (8.60 vs. 9.03, *P* > 0.05) between the the two groups. Following the intervention, the intervention group demonstrated significantly higher knowledge scores than the control group both immediately postintervention (8.55 vs. 4.54, *P* < 0.001) and 4 weeks postintervention (8.17 vs. 5.20, *P* < 0.001). Within the intervention group, knowledge scores immediate postintervention were marginally higher than those at 4-week postintervention (8.55 vs. 8.17). Similary, the intervention group achieved significantly higher practice scores than the control group at both follow-up timepoints: immediate postintervention (12.08 vs. 8.68, *P* < 0.001) and 4 weeks postintervention (11.90 vs. 8.74, *P* < 0.001). On the other hand, the mean scores of the nurses’ practical manoeuvres were slightly higher in the immediate postintervention period than at 4 weeks postintervention (12.08 vs. 11.90). In contrast, the control group showed no significant differences in knowledge or practice scores across all assessment timepoints (*all P* > 0.05). The WeChat-based educational intervention model singnificantly enhanced nurses’ knowledge acquisition and clinical practice proficiency in PIVC placement. These findings suggest that implementing such technology-driven pedagogical approaches in nursing education systems could effectively support the long-term objectives of sustaining quality improvement in clinical care and advancing professional competency development.

## Introduction

Peripheral intravenous catheter (PIVC) placement represents one of the most common procedures for establishing vascular access in clinical practice, primainly serving short-term intravenous infusion therapy to address inpatients’ needs for fluid replacement and medication administration^1–3^. This procedure is routinely performed as an invasive intervention by professionally trained nurses. Despite its widespread clinical application, PIVC use carries risks of complication during indwelling periods, such as phlebitis, accidental decannulation, catheter occlusion, thrombosis and catheter-related bloodstream infections^4–6^. These complications not only compromise therapeutic outcome but also constitute primary contributors to PIVC, and subsequent reinsertion^7^. International studies indicate that the overall incidence of PIVC failure ranges from 35 to 50%^8^, underscoring the imperative for increased clinical management and monitoring protocols aimed at reducing complications rates while improving patient safety and satisfaction.

Deficiencies in nurses’ knowledge and practice regarding PIVC management are widely recognised as key contributing factors to PIVC failure and reduced durability^9^. Evidence indicates a strong correlation between nurses’ theoretical knowledge, clinical practice and self-efficacy in PIVC insertion procedures with first-attempt success rates. Notably, procedures performed by experienced nurses demonstrate significantly lower complication rates^9,10^. Nevertheless, existing research reveals persistent gaps in nurses’ competencies across critical domains, including anatomical site selection, catheter types, determination, securement techniques, duration management, and early recognition of treatment-related complications^11,12^. In China, current strategies for mitigating PIVC complications primarily emphasize patient-centered interventions^13–15^. However, limited empirical evidence exists regarding effective approaches to enhance nurses’ professional competencies in PIVC management. This knowledge gap underscores the urgent need for rigorous exploratory studies and evidence-based practice innovations in this field.

The implementation of structured educational programs and comprehensive empowerment of nursing practice can significantly enhance nurses’ competencies in delivering holistic patient care^16–18^. In contemporary healthcare environments, the proliferation of mobile electronic devices has catalysed the evolution of nursing education methodologies. Evidence indicates that technology-enhanced learning (TEL) approaches, particularly E-learning platforms, demonstrate effectiveness not only in optimizing training efficiency and knowledge retention but also in improving nurses’ clinical competencies and educational satisfaction^19^. Compared with traditional didactic methods relying on print materials and telephonic communication, mobile-enabled learning systems provide more cost-effective solutions for content delivery. Internet-based learning further facilitates ubiquitous access to multimodal educational resources, enabling self-directed learning experiences that transcend temporal and spatial constraints. This pedagogical shift empowers learners with greater autonomy in managing their educational progression and schedule adaptation.

The primary objective of this study was to evaluate the impact of a WeChat-based continuing education program on improving nurses’ knowledge and practical skills in PIVC placement, guided by clinical guidelines. Additionally, this study aimed to systematically assess current levels of PIVC-related knowledge and clinical practices in healthcare settings, thereby establishing a foundation for future research and quality improvement initiatives. By integrating digital platforms into nursing education, this work not only advances innovative approaches to professional training but also informs the development of evidence-based strategies for enhancing clinical practice.

## Methods

### Study design and sample

This study was conducted at a tertiary general hospital. The sample size was calculated using the formula *n* = *Z*^2^*P* (1-*P*)/*e*^2^, with *Z* = 1.96 (*95% CI*l), *P* = 0.7864 (a survey report from Jiangxi Province)^20^, *e* = 0.075 (margin of error). Basis on this equation, the minimum required sample size was determined to be 115. To ensure balanced group comparisons, we allocated 120 participants to both the control and intervention groups. To account for potential attrition (e.g., dropout or incomplete data), a 20% buffer was applied, resulting in a final sample size of 72 participants per group.

To ensure the representativeness of the sample, a stratified survey method was used with three strata, namely the emergency department, medical inpatient ward and surgical inpatient ward, 24 participants were recruited within each stratum. And using the strata as a unit, the nurses were randomly allocated to the control and intervention groups by drawing lots in a 1:1 ratio.

Participants are recruited by means of announcements made by the nursing department of the hospital and by announcements made at the morning meetings of the department. Participant selection criteria were defined following: (1) possession of a valid nursing licence; (2) current clinical nursing role with PIVC placement qualifications; and (3) scoring below 75% on the baseline knowledge assessment. Exclusion criteria included: all nurses who did not work with patients for PIVC placement and those who did not agree to participate in this study were excluded. We aimed to use this design obtain reliable data to analyse in depth the performance of nurses when placing PIVCs.

### Data collection

The data collection process was conducted through a questionnaire carefully designed by the researchers. The questionnaire covered the participants’ personal characteristics, professional background, and knowledge and practice of PIVC placement. The Chinese version of the Standard Guide to Practice Standards for Intravenous Fluid Therapy (2021 edition), the Standard of Practice for Intravenous Therapy Nursing Techniques (WS/T 433–2023), and the Clinical Nursing Practice Guidelines for Common Complications of Intravenous Catheters were referenced^21^. On this basis, we synthesised the relevant domestic literature and reformulated the survey questions so that they were more in line with the actual situation in China and to ensure the validity and reliability of the data. The questionnaire was assessed for content validity by three specialist intravenous therapy nurses and one clinical nurse specialist, with a content validity index (*CVI*) of 0.89, indicating good correlation between the items and the target knowledge domains. The data collection was completed between March and September 2024. The data were collected from the following sources.

*Basic personal information*. This information included information such as age, gender, education, years of work experience, type of ward served and whether the participants had attended a workshop on PIVC placement.

*Knowledge acquisition of PIVC placement*. The participants’ knowledge was assessed by designing 10 single-choice questions. One point was awarded for correct answers to each question, and no points were awarded for incorrect answers. This assessment was designed to quantify the nurses’ level of understanding of PIVC placement knowledge and to provide a basis for subsequent education and training.

*Practical manoeuvres for PIVC placement*. A checklist was utilized to assess nurses’ performance in practical manoeuvres systematically. The scale consisted of 13 assessment items, each of which was scored by onsite observation, and the results were categorized into two categories: pass and fail. The total score for the entire checklist was 15 points, of which 1.5 points were awarded for “pass” for four items, 1 point was awarded for ‘pass’ for the remaining items, and no points were awarded for “fail”. Given the importance of repeated observations in practice assessment, to ensure objectivity and accuracy, three researchers will simultaneously conduct independent observations and score each nurse’s PIVC placement practice, and the final score will be the average of the three scores. This effectively improves the reliability and validity of the results obtained. In addition, these assessors were qualified as specialist intravenous therapy nurses or had more than 5 years of clinical infusion nursing experience, held relevant training certificates, and had independently performed ≥ 50 PIVC procedures in the past year with successful completion of a competency-based assessment.

### Intervention methods

After obtaining explicit consent from nurses in the intervention group, the research team collected their WeChat contact information during the initial study phase. Following the collection of demographic data, two to three concise educational messages regarding PIVC placement techniques were delivered through the WeChat platform twice daily (10:00 Am and 6:00 Pm) over a 10-day period. The message content was designed using cognitive load theory principles to ensure clarity and easy of comprehension. To ensure that the messages were read in a timely manner, we obtained detailed information about the Intervention group nurses’ work schedules and obtained their feedback on the messages they had read via WeChat. The educational content framework is summarized in Table 1. We did not implement any interventions for the nurses in the control group.

**Table 1:** Educational content.

Days	Content
1	PIVC basics
	Catheter selection strategies
2	PIVC placement protocols for patients of different ages (children/adults/elderly)
	Reasons for and stages of PIVC placement
3	Scientific basis and evaluation criteria for vein selection
4	Vein localisation methods based on anatomical features
5	Effective means of promoting venous filling prior to cannulation
6	Standardised preparation process for PIVC placement
7	Principles of asepsis and patient safety during tube insertion
8	Principles of asepsis and patient safety during tube insertion
9	Quality control measures for pipe mounting and pipe connection
10	Post-intubation care assessment and complication prevention programme

### Assessment methods

Prior to intervention implementation, all nurses participating in both intervention and control groups were required to complete a paper-based survey assessing PIVC placement knowledge during shift breaks, along with providing demographic information for subsequent analyses. Moreover, the research team conducted systematic observed and assessed the practical skills of nurses in both groups during real-time on-site PIVC placement operations to ensure the accuracy and reliability of the data. A postintervention assessment wasl administered immediately on the first working day following the educational intervention to evaluate immediate outcomes. To assess intervention sustainability, a follow-up assessment was performed at least four weeks after the initial evaluation to measure knowledge and skill retention.

### Ethical assessment

The study protocol received approval from the Medical Ethics Committee of Jiangxi Provincial People’s Hospital. All procedures complied with the Ethical Review of Biomedical Research Involving Human Beings issued by the China’s National Health Commission.

The purpose and importance of this study were explained in detail to the participants by the researchers, and informed consent was obtained. To ensure the autonomy of the participants, the research team assured them that they were free to withdraw from the study at any time without any negative consequences. In addition, the control group participants were provided with brief and educational poststudy information to help them grasp the knowledge and information related to the study more quickly and comprehensively. This initiative not only improved the participants’ understanding of the study content but also provided them with opportunities for further learning, thus contributing to the dissemination of scientific knowledge and the improvement of the public’s scientific literacy.

### Statistical analysis

In this study, we used SPSS 27.0 (SPSS Inc., Chicago, IL, USA) statistical software to analyse and process all the data collected systematically. First, we conducted descriptive data analysis on the participants’ basic information, using chi-square test (*χ*^2^ test) or t-tests to evaluate significant differences between different groups. Since the knowledge and practical operation scores of the nurses did not pass the normality assessment using the Kolmogorov‒Smirnov test and the Shapiro‒Wilk test, we used the Mann‒Whitney U test for comparing the scores among groups. This non-parametric test is particularly suitable for samples that do not meet the normal distribution, provide more reliable results. We set the test level at *α* = 0.05.

## Results

### Comparison of participants’ basic profiles

**Fig. 1:**
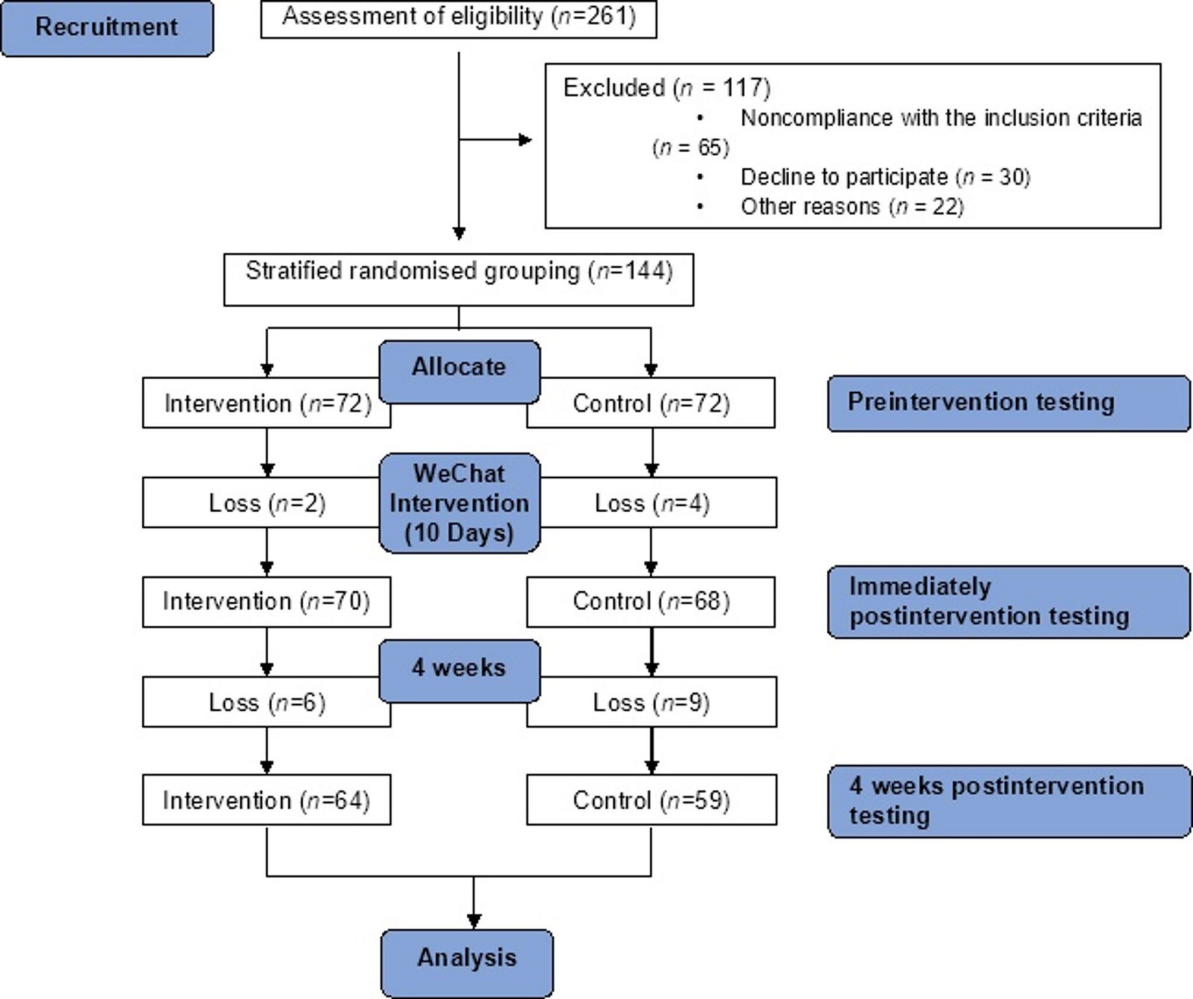
Flowchart of the study (the flowchart shows the entire process from recruitment to analysis, including participants excluded, grouped, lost and finally analysed in numbers).

A total of 123 nurses were included in this study, 64 and 59 assigned to the intervention and control groups, respectively (Fig. 1). No statistically significant differences were observed between tow groups in participants’ personal and professional characteristics (*all P* > 0.05), demonstrating baseline comparability of the groups (Table 2).

**Table 2:** Comparison of personal and professional characteristics of participants.

Characteristics	Intervention (mean ± SD) or (*n*/%)	Control (mean ± SD) or (*n*/%)	t or c^2^ Value	*P* Value
Age(y)	29.31 ± 5.13	28.15 ± 5.98	1.153	0.249
< 25	36/56.25	34/57.63	0.024	0.878
≥ 25	28/43.75	25/42.37		
Sex				
Male	4/6.25	3/5.08	–	1.000*
Female	60/93.75	56/94.92		
Education level				
Junior college	34/53.12	28/47.46	0.394	0.530
Undergraduate	30/46.88	31/52.54		
Working experience	4.67 ± 3.79	3.93 ± 4.54	1.484	0.193
< 2	31/48.44	33/59.93	0.912	0.634
2–5	16/25.00	11/18.64		
> 5	17/26.56	15/25.42		
Type of ward				
Emergency department	6/9.38	8/13.56	0.850	0.654
Internal medicine	31/48.44	30/50.85		
Surgical unit	27/42.19	21/35.59		
History of participation in workshops related to PIVC placement				
Yes	3/4.69	6/10.17	–	0.309*
No	61/95.31	53/89.83		

* Fisher exact probability

### Knowledge of PIVC placement

There was no significant difference in knowledge between the two groups of nurses preintervention (4.38 vs. 4.53, *P* > 0.05). Following implementation, the knowledge level of the intervention group was significantly higher than that of the control group both immediate postintervention (8.55 vs. 4.54) and after 4 weeks (8.17 vs. 4.71) (*all P* < 0.001). Longitudinal within-group comparisons revealed that scores in the intervention group improved by 4.17 and 3.80 points compared with baseline (*all P* < 0.001), whereas the control group demonstrated less than 0.2 points improvement. There was a small decrease of 0.38 points in the scores of the intervention group at 4 weeks postintervention compared to the immediate postintervention period, but the difference between the groups in the change over the same period compared to the control group was not statistically significant (*P* > 0.05) (Table 3).

**Table 3:** Comparison of nurses’ knowledge of PIVC placement.

Stage	Intervention	Control	Z Value	*p* Value
preintervention	4.38 ± 1.15	4.53 ± 1.09	-0.766	0.444
Immediately postintervention	8.55 ± 1.08	4.54 ± 1.10	-9.638	< 0.001
4 weeks postintervention	8.17 ± 0.81	4.71 ± 0.98	-9.686	< 0.001
Immediately postintervention vs. preintervention*	4.17 ± 1.55	0.02 ± 1.62	-8.969	< 0.001
4 weeks postintervention vs. preintervention*	3.80 ± 1.24	0.19 ± 1.40	-9.179	< 0.001
4 weeks postintervention vs. Immediately postintervention*	-0.38 ± 1.35	0.17 ± 1.62	-1.850	0.064

*Comparison by difference in individual scores between stages

### Practical manoeuvres for PIVC placement

No significant difference was observed between the PIVC placement practice scores of the two nurse groups at preintervention (8.60 vs. 9.03, *P* > 0.05). Immediately postintervention (12.08 vs. 8.68) and 4 weeks follow (11.90 vs. 8.74), the intervention group demonstrated significantly higher scores than the control group (*all P* < 0.001). Longitudinal within-group comparisons revealed increase of 3.48 and 3.30 points in the intervention group compared to preintervention levels (*all P* < 0.001), whereas the control group showed no significant score change. Although the intervention group exhibited a marginal decrease of 0.19 points at 4 weeks compared to immediate postintervention levels, the between-group difference in score changes over this period was not statistically significant (*P* > 0.05) (Table 4).

**Table 4:** Comparison of nurses’ mean scores on practical exercises for PIVC placement.

Stage	Intervention	Control	Z Value	*P* Value
Preintervention	8.60 ± 1.11	9.03 ± 1.28	-1.91	0.056
Immediately postintervention	12.08 ± 1.68	8.68 ± 0.71	-8.945	< 0.001
4 weeks postintervention	11.90 ± 1.87	8.74 ± 1.24	-7.871	< 0.001
Immediately postintervention vs. Preintervention*	3.48 ± 2.09	-0.35 ± 1.47	-8.208	< 0.001
4 weeks postintervention vs. Preintervention*	3.30 ± 2.25	-0.29 ± 1.69	-7.496	< 0.001
4 weeks postintervention vs. Immediately postintervention*	-0.19 ± 2.65	0.05 ± 1.40	-0.42	0.674

*Comparison by difference in individual scores between stages

## Discussion

This study conducted a systematic evaluation of the impact of clinical guideline education on nurses’ knowledge and practice competence in PIVC placement via a WeChat push service-based platform. The findings demonstrated that the WeChat push service-delivered educational intervention significantly enhanced nurses’ understanding and application of PIVC-related guidelines. These results not only underscore the efficacy of digital educational tools in advancing nursing expertise but also offer empirical evidence for implementing mobile platform-based educational interventions in clinical settings.

WeChat, a multifunctional social communication application developed by Tencent, has gained global popularity due to its rich features and user-friendly interface^22^. As a core functionality, the platform enables real-time communicate through text, voice, and video messaging transcending geographical barriers. This instant messaging capability not only enhances interpersonal communication efficiency but also facilitates rapid information dissemination, reshaping contemporary information acquisition and sharing patterns. In addition, the application of WeChat extends far beyond personal socialising; it also plays an important role in the fields of distance education and learning. Especially in medical education, teachers and students can establish efficient online discussions and communication platforms through WeChat, which supports real-time interaction and knowledge sharing^23–26^. Such flexible communication modalities create education opportunities that transcend the temporal and spatial constraints of traditional classroom settings.

The effectiveness of the WeChat push service could be attributed to its dual capacity for fragmented learning and immediate reinforcement. The twice-daily short message delivery aligned with nurses’ workflow patterns while enhancing memory retention (e.g. critical knowledge components including catheter selection principles and aseptic techniques) through spaced repeated. Furthermore, consistent with cognitive load theory positing that “multimodal learning enhances information processing efficiency”^23^, the incorporation of multimedia elements (text, video) likely promoted multi-channel knowledge encoding. However, a statistically significant decline in knowledge scores was observed at 4 weeks postintervention (8.55 → 8.17), indicating limitations in sustained knowledge retention through passive push-based learning modalities. This phenomenon is consistent with the Ebbinghaus forgetting curve theory, which suggests that knowledge that is not actively retrieved and practically applied tends to fade over time^24^. Notably, the practical skill scores demonstrated greater stability (12.08 → 11.90), potentially attributable to skill preservation through procedural memory mechanisms. Nevertheless, this finding underscores the insufficient of purely didactic interventions for consolidating complex clinical competencies. In the future, a hybrid model of “push + simulation training” can be explored, combining behaviourist and constructivist theories to strengthen the internalisation of skills^25^.

The control group exhibited no significant preintervention change in knowledge scores, though a minor fluctuation (0.05 points) was observed the practice scores. This variation may reflect spontaneous clinical experience accumulation or Hawthorne effects (temporary behavioral modifications due to awareness of observation). Notably, the intervention group achieved a 95.21% knowledge improvement, substantially exceeding the 31% reported in Keleekai et al.’s simulation-based study^9^. This disparity likely stems WeChat high-frequency, low-threshold deliver model, which enhances accessibility for broader populations. However, this scalability introduces trade-offs in personalisation. Junior nurses (48.44% of cohort) frequently required foundational operational guidance, while senior practitioners (26.56%) demanded advanced complication management content. The absence of stratified educational design may have compromised intervention, underscoring the necessity for career-stage-adapted content development in future implementations.

The findings of this study vaildate the applicability of the Technology Acceptance Model (TAM) in nursing education contexts^26^. The widespread adoption (> 2 h average daily usage^22^) and user-friendly interface of WeChat significantly lowered nurses’ technological resistance, whereas the clinical relevance of delivered content (e.g., catheter fixation techniques) enhanced perceived usefulness. These dual mechanisms collectively contributed to improved intervention adherence. This observation contrasts with Mousavi et al.’s^27^ SMS-based intervention demonstrating 24% skill improvenment^9^, while our video-enhanced approach achieved superior practice scores gains (40.47%). The discrepancy supports media richness theory, indicating that multimodal delivery systems incorporating video demonstrations facilitate more effective skill acquisition than text-only formats. To optimize learning outcomes, content delivery could be augmented through embedded instructional videos and synchronous consultation modules, thereby increasing both media richness and learner engagement.

This study provides empirical support for the digital transformation of nursing education. WeChat push services can be used as a low-cost and highly accessible training tool, especially in resource-limited hospitals in central and western China. However, the risk of “technology dependency” should be guarded against: the phenomenon of knowledge attrition suggests that mobile learning should be combined with institutionalised training, such as incorporating push content into the hospital’s continuing education credit system or conducting regular offline workshops to reinforce key skills. In addition, the proportion of nurses attending professional workshops was extremely low (4.69% in the intervention group), reflecting the current single form of continuing education and insufficient incentives. The policy level can promote ‘online-offline credit sharing’ and link training participation to performance appraisal to increase nurses’ learning initiative.

## Limitations

The intervention and control groups were selected from a single tertiary hospital to minimize variations in nurses’ knowledge levels attributable to differing continuing education systems across institutions. However, this design choice introduced the risk knowledge spillover between groups, as both cohorts operated within the same clinical environment, potentially compromising result validity. Furthermore, the use of non-parametric statistics prevents the generalization of the results to the population, limiting the recommendation of the intervention as a widely applicable strategy. Therefore, future research should prioritize controlling this factor to enhance methodological rigor and ensure result reliability.

## Conclusion

The WeChat push service effectively improves nurses’ short-term competence in PIVC placement through high-frequency and fragmented knowledge transfer, but its long-term effect is limited by the passive learning mode and lack of individualisation. In the future, a “push-practice-feedback” closed-loop system, combined with tiered content design and blended teaching, is needed to achieve sustainable improvement in nursing competence. This study provides localised evidence for the theory of mHealth education and suggests directions for policy makers to optimise the nursing education system.

## Data Availability

The datasets analysed in the current study are available from the corresponding author upon reasonable request.
